# Antecedents Predicting Students’ Active Use of Learning Strategies in Schools of Low SES Context within the Framework of Self-Determination Theory

**DOI:** 10.3390/ejihpe13030044

**Published:** 2023-03-06

**Authors:** Agne Brandisauskiene, Loreta Buksnyte-Marmiene, Jurate Cesnaviciene

**Affiliations:** 1 Educational Research Institute, Education Academy, Vytautas Magnus University, K. Donelaičio Str. 52, LT-44244 Kaunas, Lithuania; 2 Department of Psychology, Faculty of Social Sciences, Vytautas Magnus University, Jonavos Str. 66, LT-44191 Kaunas, Lithuania; 3 Teacher Training Institute, Education Academy, Vytautas Magnus University, K. Donelaičio Str. 52, LT-44244 Kaunas, Lithuania

**Keywords:** perceived teacher’s support, equity, perceived autonomy support, student cohesiveness, learning strategies, self-determination theory

## Abstract

Meeting a student’s autonomy, competence, and relatedness needs is one of the conditions to help him/her learn effectively. In this study, we aim to understand how teacher support (relationship with students, their autonomy support) and general classroom atmosphere (equity, relationships between students) predict students’ learning; that is, the learning strategies they use. Data were collected from 24 secondary schools in 9 municipalities in Lithuania with low SES (socioeconomic status) contexts (N = 632 students; 330 girls and 302 boys). The following instruments were used in the research: What Is Happening in this Class? (WIHIC) questionnaire, a short form of the Learning Climate Questionnaire (LCQ), and the Learning Strategies scale. The results showed that girls use learning strategies statistically significantly more actively than boys. Students’ use of learning strategies in a sample of both boys and girls are predicted by perceived teacher support, student cohesiveness, and perceived autonomy support. Therefore, responding to the relatedness and autonomy needs of students from low SES is very significant because it can increase their engagement in the learning process. The difference found, that equity is a significant predictor of learning strategies in the sample of girls, but not in boys, encourages further research and interpretation of such research results.

## 1. Introduction

The results of international achievement studies [1] show that students’ social, economic, and cultural environment has clear connections with their achievements. Students’ disadvantaged social and economic situation is a predictor of low achievement [2,3]. However, some of these students attain high academic achievement. In Australia, Canada, Estonia, Hong-Kong (China), Ireland, Macao (China), and the United Kingdom, more than 13% of disadvantaged students were academically resilient; that is, they achieved the highest quarter of learning results [1]. These results show that the school community can help students from low socioeconomic status (SES) overcome difficulties and achieve academic success. Factors related to academic resilience are parents’ and teachers’ support, school climate, and the student’s beliefs in one’s own abilities [1]. Recognizing the importance of these factors, in this study we aim to understand how teacher support (relationship with students, their autonomy support) and general classroom atmosphere (equity, relationships between students) predict students’ learning; that is, the learning strategies they use.

According to Self-Determination Theory [4], students are characterized by three basic psychological needs—autonomy, competence, and relatedness. When they are answered, students are likely to be engaged in the learning process, curious, active, and high-achieving [5]. However, it should be noted that this process is not automatic and requires support [4], so the teacher’s professionalism and understanding of the student’s needs are very important in this case. Appropriate teacher behaviour can enable effective learning for all students, including students from low SES.

Relatedness is one of the three psychological needs of students, which facilitates the process of internalization: students who feel accepted at school and experience a sense of belonging to it tend to adopt the example of school members and teachers‘ behaviour as their own; that is, internalize their values [5]. The significance of the relationship between the teacher and the student is emphasized by many other scientists, highlighting their influence in the educational process. For example, Hargreaves [6] states that students learn best when there is a safe, stable school environment and healthy relationships. Positive teacher–student relationships increase students’ engagement in the learning process [7], their learning motivation [8,9], and self-esteem [10,11]. Greater perceived support from teachers protects the student from the negative consequences of self-isolation [12] and helps the teacher to notice gaps in the student’s learning [13]. Finally, researchers [14] found that students who maintain positive relationships with teachers are more likely to have positive expectations and values related to academic success. Perhaps in this case, higher student achievement can be attributed to teacher support in reducing student stress because, according to Hughes [15], a positive relationship with the teacher allows students to direct their energy toward classroom tasks and constructive interactions with peers and other teachers.

It is clear that for students from low SES, a harmonious relationship with teachers is essential. Such a relationship can be one of the conditions that help to engage these students in the learning process [16,17]. It is likely that harmonious relationships as a sign of relatedness can also help to overcome uncertainties and tensions, which are certainly not lacking in the everyday life of students from low SES. On the other hand, it is necessary to note that not only relationships with the teacher, but also interpersonal relationships with peers are significant. A common social context that supports students’ relatedness promotes their internal learning motivation [18] and also predicts a positive experience and well-being [19]. Researchers [20] point out that the created relationship with peers can help or hinder the student’s learning success, so it is important for the teacher to see their expression. It appears that joint academic activities with a friend or friendly relationships in the classroom are associated with a better learning process and higher results [21].

From our point of view, meeting the student’s need for relatedness is also related to equity. Positive teacher–student relationships promote equity [22], mitigate the negative effect of poor performance [23], and can even protect students from delinquent behaviour in the presence of less than favourable classroom environmental conditions, e.g., not having close relationships with peers [22]. Consequently, teacher support is also a significant element of equity, and the lack of it can be especially dangerous for students who demonstrate lower learning results [24]. By purposefully creating a collaborative atmosphere in the classroom and inviting all students to be active participants in the learning process, teachers can ensure equity [25]. Namely, the promotion of equity in education is less about the introduction of particular techniques or new organizational arrangements, and much more about processes of social learning [26]. However, it is important to understand that equality is not self-evident for teachers, so their professional development is necessary in this regard [27]. General school policies and actions must be directed to improve teaching and the school learning environment [28] if equity is to be ensured for students from low SES [29].

Autonomy is another psychological need of students, the response of which is very important in the learning process [5]. Research results [30] show that autonomy support and relatedness were equally important for student achievement in both Western and Eastern cultures. Both of these needs (autonomy and relatedness) are also related and manifest together in the social, i.e., teaching and learning, context [19]. Therefore, teachers must use a motivating style that supports student autonomy rather than a controlling one [31,32]. In this way, they will meet the basic psychological needs of students and create conditions for them to feel good, be active, and strive for excellence. Researchers claim that teacher behaviour that supports student autonomy strengthens and develops students’ internal motivational resources [32], as it also provides structure and options to choose [33]. The conscious behaviour of students learning in this way is compatible with internal learning motivation, high volition, and a sense of choice over their actions [34]. In this case, it is likely that even students from low SES will be empowered for effective learning; that is, self-regulated use of learning strategies.

The quality of students’ learning is affected by learning strategies applied by themselves [35]. Learning strategies reveal how students process and assimilate information, how they manage their learning process, and what learning tools they use to achieve their goals. As stated by Melvina, Lengkanawati, and Wirza [36] (p. 63), “Learning strategies are steps taken by learners to accelerate the attainment of knowledge, the storage of that data, and retrieval of information when they are needed”. These strategies are often named as techniques used by learners to help them in the learning process [37]. The analysis of learning strategies allows to answer the questions of what makes learners successful in the learning process and why some students are more effective at learning than others [38]. Conducted studies show that successfully used learning strategies develop students’ learning autonomy, competence, and self-confidence, and increase students’ motivation and activity in the process of learning [36]. Thus, the use of learning strategies promotes students’ self-regulated learning: students become more aware of their learning process; learn to regulate their learning efforts in order to achieve their final goals; and, thus, become more and more independent [38]. It is interesting that researchers obtain different results regarding the use of learning strategies in groups of girls and boys. Some researchers claim that girls use more learning strategies [39]; others indicate that the use of strategies does not differ between groups of girls and boys [36,38]; and others find that the number of learning strategies used in groups of boys and girls do not differ, but the nature of strategies used does [40]. Thus, we tend to look at the use of learning strategies as a certain student’s competence, like a third psychological need’s expression. That is, if the student uses effective learning methods more often and more actively, this is an Important condition for mastering the skills necessary for learning and achieving high learning results.

Thus, it becomes clear that all the listed aspects—interpersonal relations with the teacher and peers, equity, autonomy-supportive behaviour, and learning strategies—are significant for an effective student learning process and can be justified by the three basic psychological needs identified in the Self-Determination Theory [4]—autonomy, competence, and relatedness. Understanding that a student’s learning strategies are his/her activities that directly describe the student’s autonomy, i.e., self-regulated learning, and can guarantee good learning results, in this study, we propose a hypothesis: the student’s mutual relations with the teacher and peers, equity, and perceived autonomy support predict more active use of learning strategies applied by students.

## 2. Materials and Methods

### 2.1. Sample and Procedure

An invitation to participate in the study was sent to 54 Lithuanian general education schools located in small towns or rural areas [41] (p. 33). These are schools with a small number of students and a low social, economic, and cultural context. Between 30% and 40% of pupils in these schools receive free school meals (the use of eligibility for a free lunch is a measure of a student’s low socioeconomic status). Twenty-four general education schools accepted the invitation. After informed consent was gained from school principals, data collection took place in May 2021. Only students who received parental permission participated in the study and voluntarily completed the self-report anonymous questionnaire on the online platform, https://apklausa.lt (accessed on 31 May 2021). The research sample was composed of 632 students (330 girls and 302 boys). The students were enrolled from seventh (21.8%), eighth (20.1%), ninth (23.7%), and tenth (34.3%) grades. The pupils’ ages ranged from 13 to 16 years; hence, these students were in a formal education programme (lower secondary education), which is compulsory in Lithuania until the age of 16.

### 2.2. Instruments

In this study, we will measure the expression of the student’s relationship with teachers, peers, and equity with three subscales from What Is Happening in this Class? (WIHIC) [42]. In the Student Cohesiveness subscale, we will measure students’ relationships with their peers because it evaluates the extent to which students are friendly and supportive of each other (e.g., this teacher talks with me). The Teacher Support subscale (e.g., the teacher considers my feelings) describes students’ perception to which extent the teacher helps, befriends, and is interested in students, so using it we will see the expression of personal student–teacher relationships. The Equity subscale (e.g., I get the same opportunity to answer questions as other students) identifies the extent to which the teacher treats students equally, including distributing praise, questions, and opportunities to be included in discussions. Each item employs a 5-point Likert response format (from 1—almost never to 5—almost always). The KMO index (0.956) and Bartlett’s test of sphericity (χ² = 11645.544, *p* < 0.001) indicated that the data were suitable for factor analysis. Factor analysis indicated three factors with eigenvalues greater than 1.00, which accounted for 66.9% of the total variance. Factor loadings ranged from 0.414 to 0.807. Cronbach’s alpha for the three subscales were 0.927, 0.913, and 0.928, respectively. McDonald’s omega was 0.928, 0.914, and 0.928, respectively.

The Learning Climate Questionnaire [43] is chosen to measure students’ response to the need for autonomy. The short form of this questionnaire measured how students perceive the autonomy support provided by their teachers. This questionnaire consisted of 6 items (e.g., I feel that my teacher provides me choices and options) answered on a 7-point Likert scale from 1 (strongly disagree) to 7 (strongly agree). The KMO index (0.875) and Bartlett’s test of sphericity (χ² = 3092.001, *p* < 0.001) indicated that the data were suitable for factor analysis. The one-factor measurement model explained 73.2% of the total variance, with factor loadings ranging from 0.813 to 0.894. The overall Cronbach’s alpha and McDonald’s omega were 0.926 and 0.924, respectively. Students’ learning strategies were measured by the Learning Strategies scale. Statements for the scale were formulated by the first authors of the article. The scale consists of nine self-report Likert scale items (e.g., I use a variety of techniques (such as repeating orally, doing diagrams) to memorize the information I need), ranging from 1 (almost never) to 5 (almost always). The internal consistency reliability of the scale as estimated by Cronbach’s alpha and McDonald’s omega was reasonably high at 0.905. The KMO index (0.928) and Bartlett’s test of sphericity (χ² = 2904.941, *p* < 0.001) indicated that the data were suitable for factor analysis. The one-factor measurement model explained 57.3% of the total variance. Item loadings from a principal factor analysis with Varimax rotation are presented in Table 1.

### 2.3. Data Analysis

The statistical analyses in the study were conducted with the IBM SPSS Statistics 26.0. The degree of normality of continuous variables’ distributions was checked by skewness and kurtosis. Parametric statistics were calculated for variables with skewness <−1 or >1 and kurtosis <−3 or >3. To examine the factorial validity of the research instruments, an exploratory factor analysis was carried out using the principal factor extraction method and Varimax rotation. We used the Cronbach’s alpha and McDonald’s omega to test the internal consistency and reliability of the questionnaire. These coefficients of 0.70 or higher for a set of items were considered acceptable [44].

First, descriptive statistics were run on the independent (student cohesiveness, perceived teacher support, equity, and perceived autonomy support) and dependent variables (learning strategies applied by students) used in the study to determine means and standard deviations. Additionally, bivariate correlation analysis of all variables was performed using Pearson’s correlation coefficients, wherein r = 0.10–0.29 was a small correlation, r = 0.30–0.49 a moderate correlation, and r ≥ 0.5 a strong correlation. Secondly, the multivariate analysis of variance (MANOVA) was calculated to analyse the differences of variables by gender. Effect sizes (Partial Eta Squared Coefficient) were calculated for the interaction effects, with an effect size of η^2^ = 0.01 representing a small effect, η^2^ = 0.06 representing a medium effect, and ηp^2^ = 0.14 representing a large effect [44]. Next, a multiple linear regression was performed separately for the girls’ sample and boys’ sample. Pearson’s correlation coefficient and variance inflation factor (VIF) helps to check the multicollinearity of independent variables. High correlation values (greater than 0.8) and a VIF score of four or above indicate multicollinearity [45]. In regression analysis, the effect size of the predictor variables is given by the beta loadings. In interpreting the effect, size gives the following guidance: 0–0.1 = weak effect, 0.1–0.3 = modest effect, 0.3–0.5 = moderate effect, and >0.5 = strong effect [44]. An alpha level of 0.05 was used as a significance level for all the statistical analyses.

## 3. Results

The descriptive statistics and intercorrelations between all variables in the study are outlined in Table 2. From the analysis of the skewness (−0.608 to −0.030) and kurtosis (−0.590 to 0.243) values, it is established that the research data were close to a normal distribution. An examination of the means of the variables revealed that the highest scores were students’ perceived autonomy support (M = 4.39), and the lowest average score was ascribed to perceived teacher support (M = 3.06). The results of bivariate Pearson’s correlations indicated that the dependent variable (learning strategies applied by students) correlated positively and significantly to the independent variables (r = 0.483, *p* < 0.01 for the student cohesiveness; r = 0.572, *p* < 0.01 for the perceived teacher support; r = 0.552, *p* < 0.01 for the equity; and r = 0.494, *p* < 0.01 for the perceived autonomy support). The correlations between independent variables revealed that absolute values of Pearson r coefficients are less than 0.8; this indicates that multicollinearity is very unlikely to exist.

We then ran MANOVA with student gender as an independent variable and the five dependent variables, namely student cohesiveness, perceived teacher support, equity, perceived autonomy support, and learning strategies applied by students. The Box’s M test (Box M = 10.64, *p*  =  0,784) was not significant; thus, the observed covariance matrices of the dependent variables are equal across groups. The results of the MANOVA showed that there was a statistically significant difference between the girls and boys on the combined de-pendent variables F  =  4.295 b, *p*  ≤ 0.001, Wilk’s Lambda  =  0.033. The obtained results (Table 3) show that the averages of the three independent variables of the study (student cohesiveness, perceived teacher support, and perceived autonomy support) do not differ statistically significantly in the sample of boys and girls. However, the mean of the fourth independent variable (equity) in the sample of girls (M = 3.75, SD = 0.90) is higher than that of boys (M = 3.55, SD = 0.92). A statistically significant difference was obtained (F = 7.488, *p* = 0.006), although partial eta squared (η^2^ = 0.012) shows a small effect.

It was also found that the averages of the dependent variable (learning strategies applied by students) are statistically significantly different (F = 16.128, *p* = 0.0001): in the sample of girls (M = 3.22, SD = 0.79), it is higher than that of boys (M = 2.97, SD = 0.77). Although there is a difference, η^2^ = 0.025 shows a small effect.

A multiple linear regression was conducted to determine if the independent variables (student cohesiveness, perceived teacher support, equity, and perceived autonomy support) collectively predict the dependent variable (learning strategies applied by students). Since the means of the dependent variable are statistically significantly different, the regression analysis was performed separately for the sample of boys and the sample of girls.

The first multiple linear regression analysis included the boys sample (n= 302). All four independent variables were initially included in the model. However, when included in multiple regression analyses, equity as predictor was marked as not significant (*p* = 0.269). Therefore, the multiple regression analysis was repeated. The results of the improved model are presented in Table 4. The R^2^ = 0.409 shows that 40.9% of the dependent variable can be predicted by the independent variables (F = 68.722, *p* < 0.0001). A detailed analysis of the β coefficients showed that the perceived teacher support (β = 0.335) was the best predictor of learning strategies applied by students (t = 5.665, *p* = 0.0001). The other two predictors are weaker.

The second multiple linear regression analysis included the girls sample (n = 330). All four independent variables were statistically significant (Table 5). Perceived teacher support had a stronger predictive effect for girls’ when predicting learning strategies (β = 0.318), compared to equity (β = 0.173), student cohesiveness (β = 0.145), and perceived autonomy support (β = 0.119). A significant regression model was found (F = 53.704, *p* = 0.0001), with an R^2^ of 0.398. This means that 39.8% of learning strategies applied by girls can be explained by all four predictors.

## 4. Discussion

Learning strategies applied by the students are an important antecedent of students’ learning performance and satisfaction [35,46], so it is important to study the factors of the learning environment that determine the more active use of learning strategies. The results of the conducted research allow us to provide several important insights. It becomes clear that girls and boys do not equally actively apply learning strategies: girls use them more actively than boys and this difference is statistically significant. Since the use of learning strategies can be associated with engagement in learning [47], this research result is corroborated by the data of other researchers. For example, a large group of researchers [48] see a consistent trend of higher engagement in learning for girls than boys in as many as 12 countries. In 2022, the published UNESCO report “Leave no child behind: global report on boys’ disengagement from education” [49] also shows that, according to data from many countries, boys are more at risk than girls of not being active learners and achieving poorer academic results at school. Therefore, it is obvious that it is necessary to look for factors that could encourage all students (and boys in particular) to become more and more actively involved in the learning process, and the results of our research provide certain answers.

The factors that determine the learning process and, accordingly, the achievement of boys and girls are many and varied. According to Cascella et al. [50], they can be individual, social, and cultural, as well as factors related to the school context, such as curriculum, teaching practices in the classroom, and teacher evaluation methods [51]. It seems that even the gender of the teacher can make a difference. For example, some researchers [52] claim that male and female teachers perceive and evaluate male and female students differently. Theoretically, teachers may favour students who are more similar to themselves and, consequently, give them higher evaluations [53]. On the other hand, a teacher of the same gender can serve as a role model [54], and, thus, a teacher of the same gender can influence a student‘s effort [55]. Lowe and colleagues [56] state that the gender of the teacher is an important factor and that girls respond more strongly than boys to same-gender role models. Therefore, the result of our study is quite clear: that in the studied sample, girls were more involved in the learning process; that is, they used learning strategies more actively. However, it is necessary to mention that research does not provide unequivocal answers as to whether there is a causal relationship between a same-gender teacher and student achievement because, as already mentioned earlier, the student’s learning process can be influenced by various factors [53].

The regression analysis carried out in our study reveals that the satisfaction of relatedness and autonomy needs is very important for students of both genders. In the sample of boys, even 40.9% of the dependent variable (that is, the use of learning strategies) predicts student cohesiveness, perceived teacher support, and perceived autonomy support. In the sample of girls, 39.8% of learning strategies applied by girls are explained by four predictors: perceived teacher support, equity, student cohesiveness, and perceived autonomy support. Thus, the results of this study confirm the idea of other researchers that factors promoting relatedness such as peer learning, working in peers’ groups, good relationship with the teacher, and the teacher’s support encourage more active use of learning strategies [35]. On the other hand, it is important for teachers to support students’ autonomy because thanks to it, students’ self-regulated learning is developed, which, in turn, promotes more active use of learning strategies, and can support students to learn independently inside and outside the classroom [36].

It is noteworthy that perceived teacher support is the best predictor of learning strategies applied by boys, when the other two predictors (student cohesiveness and perceived autonomy support) are weaker. Thus, it is clear that a close relationship between the teacher and a boy-student is necessary, which is confirmed by other researchers [57]. It is evident that perceived teacher support also has the strongest predictive effect for girls, whereas the other three variables (equity, student cohesiveness, and perceived autonomy support) have less. Hargreaves [6] states that healthy relationships between teachers and students, when attention is paid to the social–emotional aspects of interaction, promote student learning. Research by other researchers [3,48] also show that teacher support is significant: it predicts class and school-related interest, greater motivation for learning, students’ engagement in learning, and better achievement. It is claimed that teachers’ support is particularly important in adolescence and can act as a protective factor in the learning process [58].

We would like to point out one more difference that our research results show. In the sample of girls, equity is a significant predictor of learning strategies; that is, it seems that for girls’ more active use of learning strategies, it is important how teachers create an atmosphere of cooperation in the classroom by generating equal learning opportunities for every student, involving all students in an active learning process [25]. However, in the sample of boys, equity is not a significant factor in the active use of learning strategies. It must be admitted that such a result of the study is somewhat unexpected. Perhaps one of the possible explanations for such an unexpected result could be the claims of other scientists [59] that teachers are important starting points for promoting gender equality in education, as their attitudes and instructional practices influence students’ performance. A certain favouritism of the teacher towards the student of the same gender is expected [53] because according to the data of our study, girls experience a higher expression of equity and this is a significant predictor of learning strategies; hence, the respect to diversity is one of the most significant values in the relationship between teachers and students [25], and teachers have to reflect on their own gender stereotypes [59].

Thus, the results of the research show that boys are less active in the learning process than girls, and the use of learning strategies by students of both genders predicts perceived teacher support, student cohesiveness, and perceived autonomy support; hence, meeting students’ relatedness and autonomy needs is very significant, as it can increase students’ engagement in the learning process [4,34,60]. Relatedness contributed strongly to the autonomous motivation [61], and relatedness and autonomy not only do not contradict each other or are opposites, but are closely related [19]. Finally, we want to emphasize once again the significance of teacher support as a personal emotional relationship between teacher and student for the successful learning process of a student from low SES. It becomes evident that the school community can help students from low SES to be more involved in learning and academical resilience if teachers provide support and create a favourable school learning environment [1,28].

It is also necessary to discuss the limitations of this study. First, our research design was cross-sectional. This makes it possible to evaluate the correlations between different factors, but does not allow a deeper analysis of how the inclination to more actively use learning strategies is formed. Therefore, the longitudinal research design for future studies would be preferable. Secondly, the self-report method was used in this study: students themselves evaluated teacher’s support, autonomy support, and equity. In order to obtain more objective data about the significance of the researched factors for learning strategies applied by the students in future studies, it would be useful to use more diverse research data collection sources (e.g., not only from the students, but also teachers, evaluations of independent observers, etc.).

## 5. Conclusions

Understanding students’ needs and professional teacher behaviour can enable effective learning for all students, including students from low SES. The results of a study conducted in small towns or rural areas of Lithuania with a low social, economic, and cultural context show that boys in grades 7–10 use learning strategies less actively than girls. This use of learning strategies by students of both genders predicts perceived teacher support, student cohesiveness, and perceived autonomy support. Therefore, responding to the relatedness and autonomy needs of students from low SES is very significant because it can increase their involvement in the learning process. The difference found, that equity is a significant predictor of learning strategies in the sample of girls, but not in boys, encourages further research and interpretation of such research results.

## Figures and Tables

**Table 1:** Results of exploratory factor analysis for the Learning Strategies scale.

Learning Strategies Scale	Factor Loadings	Item-Total Correlation	Cronbach’s Alpha if Item Deleted
I use a variety of techniques (e.g., verbal repetition, diagramming, etc.) to remember the necessary information.	0.745	0.664	0.895
While studying at home, I repeat (information) many times	0.787	0.716	0.891
I explain what I learn to my friends	0.710	0.635	0.897
I listen intently as teachers re-explain more difficult topics over multiple lessons	0.719	0.632	0.897
I come up with different techniques to study the same topic	0.792	0.724	0.890
While studying at home, I relate new material to what is already known	0.780	0.706	0.892
I learn actively when we do more difficult tasks of a topic in several lessons	0.816	0.749	0.889
While studying, I compare the material, find similarities and differences between phenomena	0.813	0.744	0.889
While studying, I remember funny, entertaining stories related to the learning material	0.633	0.550	0.904
Mean (SD)	3.10 (0.79)		
Eigenvalue	5.158		
% Variance	57.3		
Cumulative % variance	57.3		

**Table 2:** Descriptive statistics and Pearson r correlation coefficients.

	M	SD	Skewness	Kurtosis	1	2	3	4	5
1. Student cohesiveness	3.70	0.89	−0.608	0.022	-				
2. Perceived teacher support	3.06	0.86	−0.103	−0.161	0.496 **	-			
3. Equity	3.66	0.92	−0.528	−0.108	0.749 **	0.653 **	-		
4. Perceived autonomy support	4.39	1.45	−0.306	−0.590	0.442 **	0.621 **	0.577 **	-	
5. Learning strategies applied by students	3.10	0.79	−0.030	0.243	0.483 **	0.572 **	0.552 **	0.494 **	-

Note: ** *p* < 0.01.

**Table 3:** MANOVA results with means and standard deviations for the variables on both groups (boys and girls).

					MANOVA Test		Partial Eta Squared
		M	SD	Mean Square	F	*p*-Value	
Student cohesiveness	Boys	3.64	0.91	2.068	2.567	0.110	0.004
	Girls	3.76	0.89				
Perceived teacher support	Boys	3.01	0.85	1.297	1.763	0.185	0.003
	Girls	3.11	0.86				
Equity	Boys	3.55	0.92	6.241	7.488	0.006	0.012
	Girls	3.75	0.90				
Perceived autonomy support	Boys	4.33	1.47	2.595	1.234	0.267	0.002
	Girls	4.45	1.43				
Learning strategies applied by students	Boys	2.97	0.77	9.894	16.128	0.0001	0.025
	Girls	3.22	0.79				

**Table 4:** Multiple linear regression with learning strategies applied by students as the dependent variable (boys’ sample).

	Unstandardised Coefficients B	95% CI for B	Standardised Coefficients β	t	*p*-Value	VIF
Constant	0.917	0.613 to 1.221		5.944	0.0001	
Student cohesiveness	0.179	0.088 to 0.269	0.210	3.896	0.0001	1.469
Perceived teacher support	0.304	0.198 to 0.409	0.335	5.665	0.0001	1.760
Perceived autonomy support	0.113	0.053 to 0.173	0.216	3.694	0.0001	1.722

**Table 5:** Multiple linear regression with learning strategies applied by students as the dependent variable (girls’ sample).

	Unstandardised Coefficients B	95% CI for B	Standardised Coefficients β	t	*p*-Value	VIF
Constant	0.968	0.645 to 1.291		5.900	0.0001	
Student cohesiveness	0.129	0.016 to 0.243	0.145	2.247	0.025	2.244
Perceived teacher support	0.292	0.180 to 0.404	0.318	5.130	0.0001	2.071
Equity	0.152	0.024 to 0.280	0.173	2.338	0.020	2.948
Perceived autonomy support	0.066	0.004 to 0.128	0.119	2.095	0.037	1.736

## Data Availability

The datasets generated and analysed during the current study are not publicly available due to privacy and ethical concerns, but are available from the corresponding author on reasonable request.
